# Efficacy of Short-Term Antiarrhythmic Drugs Use after Catheter Ablation of Atrial Fibrillation—A Systematic Review with Meta-Analyses and Trial Sequential Analyses of Randomized Controlled Trials

**DOI:** 10.1371/journal.pone.0156121

**Published:** 2016-05-25

**Authors:** Weijie Chen, Hang Liu, Zhiyu Ling, Yanping Xu, Jinqi Fan, Huaan Du, Peilin Xiao, Li Su, Zengzhang Liu, Xianbin Lan, Bernhard Zrenner, Yuehui Yin

**Affiliations:** 1 Department of Cardiology, the Second Affiliated Hospital of Chongqing Medical University, Chongqing, China; 2 MedizinischeKlinik I (B.Z.), Krankenhaus Landshut/Achdorf, Landshut, Germany; University of Tampere, FINLAND

## Abstract

**Background:**

The efficacy of short-term antiarrhythmic drugs (AADs) use compared with no-AADs prescription after catheter ablation of atrial fibrillation (AF) in preventing atrial arrhythmia recurrence is uncertain.

**Methods:**

We searched PubMed, Embase, and the Cochrane Library through December 2015 to identify randomized controlled trials (RCTs) which evaluated the efficacy of short-term AADs use compared with no-AADs prescription after AF ablation in preventing atrial arrhythmia recurrence. The primary outcome was labeled as early atrial arrhythmia recurrence within 3 months after ablation. Secondary outcome was defined as late recurrence after 3 months of ablation. Random-effects model or fixed-effects model was used to estimate relative risks (RRs) with 95% confidence intervals (CIs).

**Results:**

Six RCTs with 2,667 patients were included into this meta-analysis. Compared with no-AADs administration after AF ablation, short-term AADs use was associated with significant reduction of early atrial arrhythmia recurrence (RR, 0.68; 95% CI, 0.52–0.87; p = 0.003). Trial sequential analysis (TSA) showed that the cumulative Z-curve crossed the trial sequential monitoring boundary for benefit, establishing sufficient and conclusive evidence. However, compared with no-AADs prescription, short-term AADs use after AF ablation didn’t significantly reduce the risk of late atrial arrhythmia recurrence (RR, 0.92; 95% CI, 0.83–1.03; p = 0.15). TSA supported this result; meanwhile the estimated required information size (1,486 patients) was also met.

**Conclusion:**

Short-term use of AADs after AF ablation can significantly decrease the risk of early atrial arrhythmia recurrence but not lead to corresponding reduction in risk of late atrial arrhythmia recurrence.

## Introduction

Arial fibrillation (AF), predisposing patients to an increased risk of stroke, heart failure and death, is the most common arrhythmia encountered in clinical practice[1,2]. A key therapeutic goal of AF is to restore and maintain sinus rhythm. Catheter ablation, as a guideline-supported treatment option to restore sinus rhythm for patients with drug refractory AF, is increasingly performed in routine clinical practice[1].

However, the recurrences of AF or other atrial arrhythmia following catheter ablation are very common, estimated up to 50% after a single intervention[3,4,5,6,7]. Referred to early recurrence of atrial arrhythmias, one of the most prominent causes is the transient post-ablation inflammation following the histopathologic tissue damage that resulted from RF energy, which contributes to the heterogeneity of action potential durations in the myocardium of the atrial and/or pulmonary veins by inflammatory cytokines and ultimately results in the creation of an arrhythmogenic substrate that may be related to the initiation of AF[8,9,10,11]. It is known that antiarrhythmic drugs (AADs), especially the class I and III AADs, could lead to the prolongation of the effective refractory period and the action potential durations of atrial myocytes by blocking the sodium and potassium channels. Therefore, theoretically, the use of AADs after catheter ablation of AF may prevent the early recurrence of atrial arrhythmias through improving the heterogeneity of action potential durations of the atrial myocardium. Moreover, early recurrence of atrial arrhythmias, although not a predictor of treatment failure in AF patients who received catheter ablation[1,12], has been shown to be a strong indicator of late recurrence[13,14,15]. Thus, pharmacologic rhythm control approach with short-term use of AADs within 3 months after ablation has been proposed by the guidelines and expert consensus[1,12]. Short-term use of AADs is also often prescribed by experience in clinical practice.

However, the efficacy of AADs short-term use after catheter ablation of AF was just investigated in several small size randomized controlled trials (RCTs) [13,16,17], each of which enrolled only 50–200 patients until the results of a large-scale multicentre study with 2038 patients were published on European Heart Journal in October 2015[14].

Evidence from the above RCTs reported inconsistent results. And a published small-size meta-analysis conducted by Xu et al[18] was underpowered to reach determinate conclusions because of the limitations that need to be addressed: first, the included patients in the meta-analysis had obvious clinical heterogeneity, as the included trial by Brignole et al[19] involved AF patients received atrioventricular junction ablation but not PVI-based catheter ablation, and in another included RCT by Jun et al[20] the control group received either class I or III AADs but not placebo while the extensive AADs therapy group received both class I and III AADs. Second, apart from these two above mentioned RCTs with clinical heterogeneity, only 4 RCTs with 555 patients had the appropriate clinical homogeneity for meta-analysis. Thus, we conducted a meta-analysis of the latest and most convincing evidence to evaluate the efficacy of short-term AADs use compared with no-AADs after catheter ablation of AF in preventing atrial arrhythmia recurrence; and we further applied trial sequential analysis (TSA) to determine whether the currently available evidence was sufficient and conclusive.

## Methods

This meta-analysis was performed according to the recommendations of the *Cochrane Handbook for Systematic Reviews of Interventions*[21] and was reported in compliance with the PRISMA (Preferred Reporting Items for Systematic Reviews and Meta-Analyses statement) guidelines[22]. There was no registered protocol for this meta-analysis.

### Literature search strategy

We searched the published literature in PubMed, Embase and the Cochrane Library from inception through December 27, 2015. The systematic electronic searches were conducted using exploded Medical Subject Headings (MeSH) terms and the corresponding keywords in Title/abstract. The search terms used in this meta-analysis were (MeSH exp ‘Atrial Fibrillation’, and keywords ‘atrial’, ‘atrium’, ‘auricular’, ‘fibrillation’, ‘fibrillations’,), (MeSH exp ‘Anti-Arrhythmia Agents’, and keywords ‘anti-arrhythm*’, ‘antiarrhythm*’, ‘antifibri*’), and (MeSH exp ‘Catheter Ablation’ and keywords ‘ablation’,). No language restriction was applied. To ensure literature saturation, we re-ran the searches on December 31, 2015. We also searched ClinicalTrials.gov registry (https://clinicaltrials.gov/) and manually checked the reference lists of previous published reviews and included trials to identify other potentially eligible trials. Two reviewers (W.C. and H.L.) independently conducted the initial search, deleted duplicate records, screened the titles and abstracts for relevance, and identified records as included, excluded or uncertain. In case of uncertainty, full-text article was acquired to identify eligibility. Doubts and disagreements were solved by discussion and consensus.

### Selection criteria

Published RCTs meeting the following criteria were included: (1) Population: AF patients undergoing catheter ablation with pulmonary vein isolation (PVI)-based strategy; (2) Intervention: short-term administration of AADs within 3 months after ablation of AF; (3) Comparison: no-AADs prescription after ablation procedure; and (4) Outcome: early recurrence of atrial arrhythmia lasting more than 30 s within the first 3 months after ablation, late recurrence of atrial arrhythmia lasting more than 30 s post the 3 months’ “blank period” after ablation.

### Data extraction

Data extraction was conducted by Weijie Chen, and independently confirmed by other authors (Z.L. and Y.Y.). The following information was obtained using a standardized Excel (Microsoft Corporation) data extraction form: first author, year of publication, country, study population (AF type, AF duration), number of patients, demographics of patients, echocardiographic parameters (left ventricular ejection fraction, left atrial dimension), comorbidities of patients, ablation procedural data (ablation strategy, ablation procedural endpoints) and outcomes data (follow-up duration, study endpoints, AADs in antiarrhythmic group, therapeutic strategy in control group, anti-arrhythmia duration after ablation, early recurrent events, early cardioversion events, and late recurrent events). Additionally, we also reviewed supplementary appendices of included RCTs and contacted the corresponding authors to verify extracted data and request the unreachable data, if necessary. Discrepancies during data extraction were resolved by discussion with co-authors. Predefined primary outcome was early recurrence of atrial arrhythmia within the first 3 months after ablation and the secondary outcome was late recurrence of atrial arrhythmia.

### Risk of Bias Assessment

Risk of bias was independently assessed by two reviewers (W.C. and Y.X.) using the Cochrane risk-of-bias tool[23]. According to the tool, each included trial was reviewed and scored as ‘high’, ‘low’, or ‘unclear’ risk with the following criteria: random sequence generation, allocation concealment, blinding of participants and personnel, blinding of outcome assessment, incomplete outcome data, selective reporting and other bias. Trials with high risk of bias for any domain were considered as at high risk of bias; while trials with low risk of bias for all key domains were considered as at low risk of bias; otherwise they were considered as at unclear risk of bias.

### Grading Quality of Evidence

The quality of evidence for primary and secondary outcomes of this meta-analysis was independently evaluated by two reviewers (W.C. and J.F.) according to the GRADE methodology for risk of bias, inconsistency, indirectness, imprecision, and publication bias; and classified as very low, low, moderate, or high. Summary tables were constructed using the GRADE Profiler (GRADEpro, version 3.6.1)[24].

### Statistical Analysis

Statistical analyses were performed using Stata 13.0 (StataCorp LP, College Station, Texas) and Review Manager 5.3. (The Nordic Cochrane Centre, The Cochrane Collaboration, 2014). Relative risks (RRs) with 95% CIs were calculated for dichotomous outcomes. Heterogeneity across studies was quantified using the I^2^ statistic[25]. Studies with an I^2^ statistic of 25% to 50% were considered to have low heterogeneity, those with an I^2^ statistic of 50% to 75% were considered to have moderate heterogeneity, and those > 75% were considered to have a high degree of heterogeneity[25]. An I^2^ value greater than 50% indicates significant heterogeneity, and the Mantel-Haenszel method with random effects model was used to calculate pooled RRs and 95% CIs; otherwise, the fixed effects model was used. For the meta-analyses with significant heterogeneity, sensitivity analysis was performed to evaluate the influence of single studies on the summary estimates and the consistency of the outcome. Because graphical evaluation with funnel plot can be subjective, we performed both Harbord[26] and Peters[27] tests, as formal statistical tests for publication bias. P value < 0.05 was considered statistically significant, except where otherwise specified such as p < 0.1 indicated statistically significant for heterogeneity test and publication bias evaluation with Harbord and Peters tests.

In meta-analysis, random errors because of sparse data and repetitive testing of accumulating data increase the risk of type I error[28,29]. To avoid this and correct for the increased risk of random errors, trial sequential analysis (TSA) was recommended to be used, which can determine whether the evidence in a meta-analysis is reliable and conclusive[28,30]. TSA is a methodology that combines an estimated required information size calculation for a meta-analysis with the adaptation of monitoring boundaries to evaluate the accumulated evidence. Similar to a sample size calculation for a single trial, the estimated required information size calculation involves a methodology that includes type I error, type II error, the control event proportion, and the effect size, allowing a quantification of the reliability of cumulative data in meta-analyses. Based on the theory of TSA, when the cumulative Z-curve crosses the trial sequential monitoring boundary or enters the futility area, a sufficient level of evidence for the anticipated intervention effect may have been reached and no further trials are needed. If the Z-curve does not cross any of the boundaries and the required information size has not been reached, evidence to reach a conclusion is insufficient. For our TSAs in this review, the required information size was estimated using α = 0.05 (two sided), β = 0.20 (power 80%), the control event proportions calculated from the control group of this meta-analysis, and an estimated relative risk reduction of 25% in early recurrence of atrial arrhythmia and 20% in late recurrence of atrial arrhythmia. All these trial sequential analyses were performed using software TSA version 0.9 beta (http://www.ctu.dk/tsa, Copenhagen Trial Unit, 2011)[31].

## Results

### Literature search

**Fig 1:**
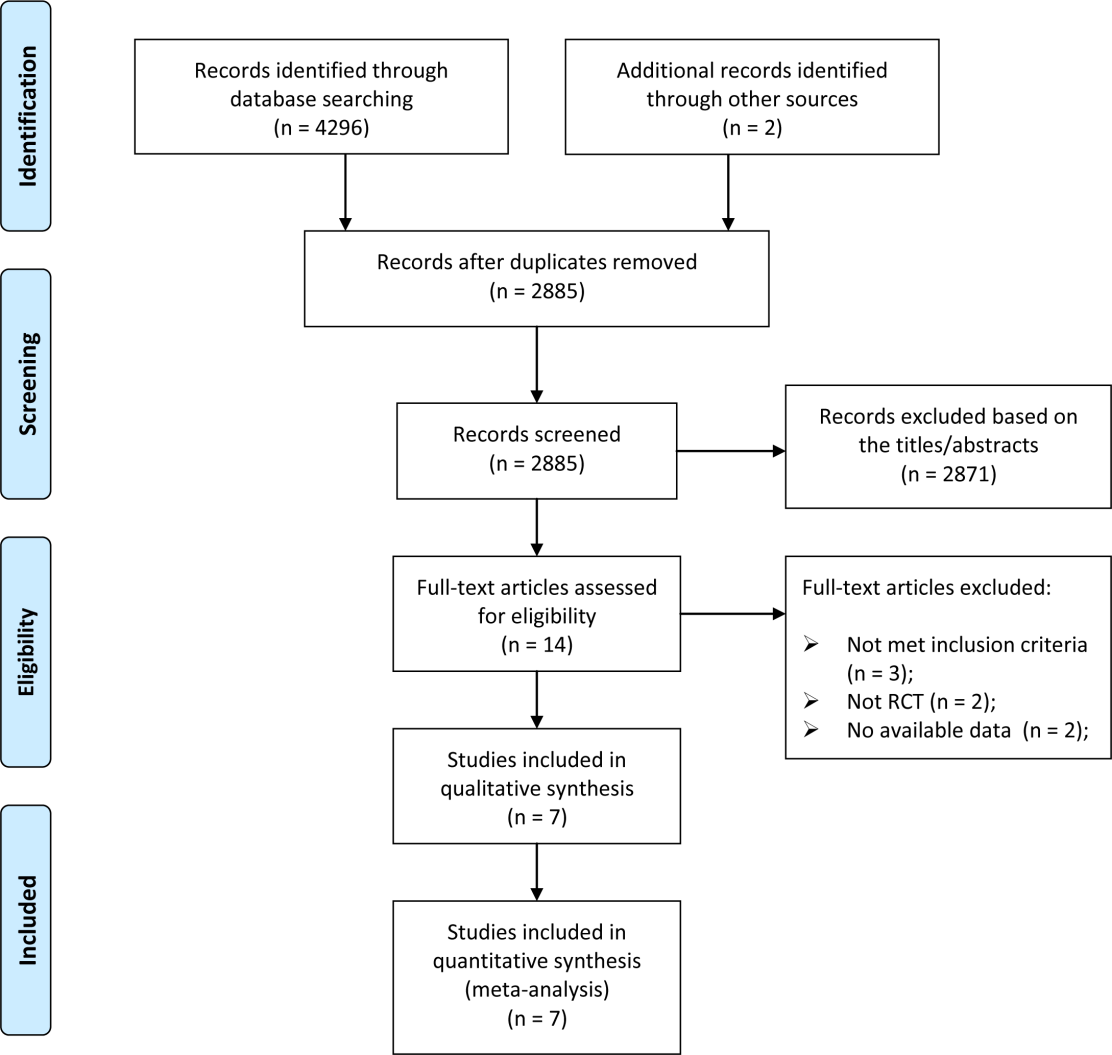
Selection of randomized controlled trials for this meta-analysis. RCT = randomized controlled trials.

The results of literature search and selection are shown in the PRISMA flowchart (**Fig 1**). Our initial search yielded 4298 records. After removing duplicates and screening the titles and abstracts, 14 articles were thought to be potentially eligible for inclusion. After full-text review, 7 published reports[13,14,16,17,32,33,34] with the results from 6 RCTs were finally included in this meta-analysis. Two of the published reports were results of the first 6 weeks and 6 months related to the same study.

### Trials Characteristics

**Fig 2:**
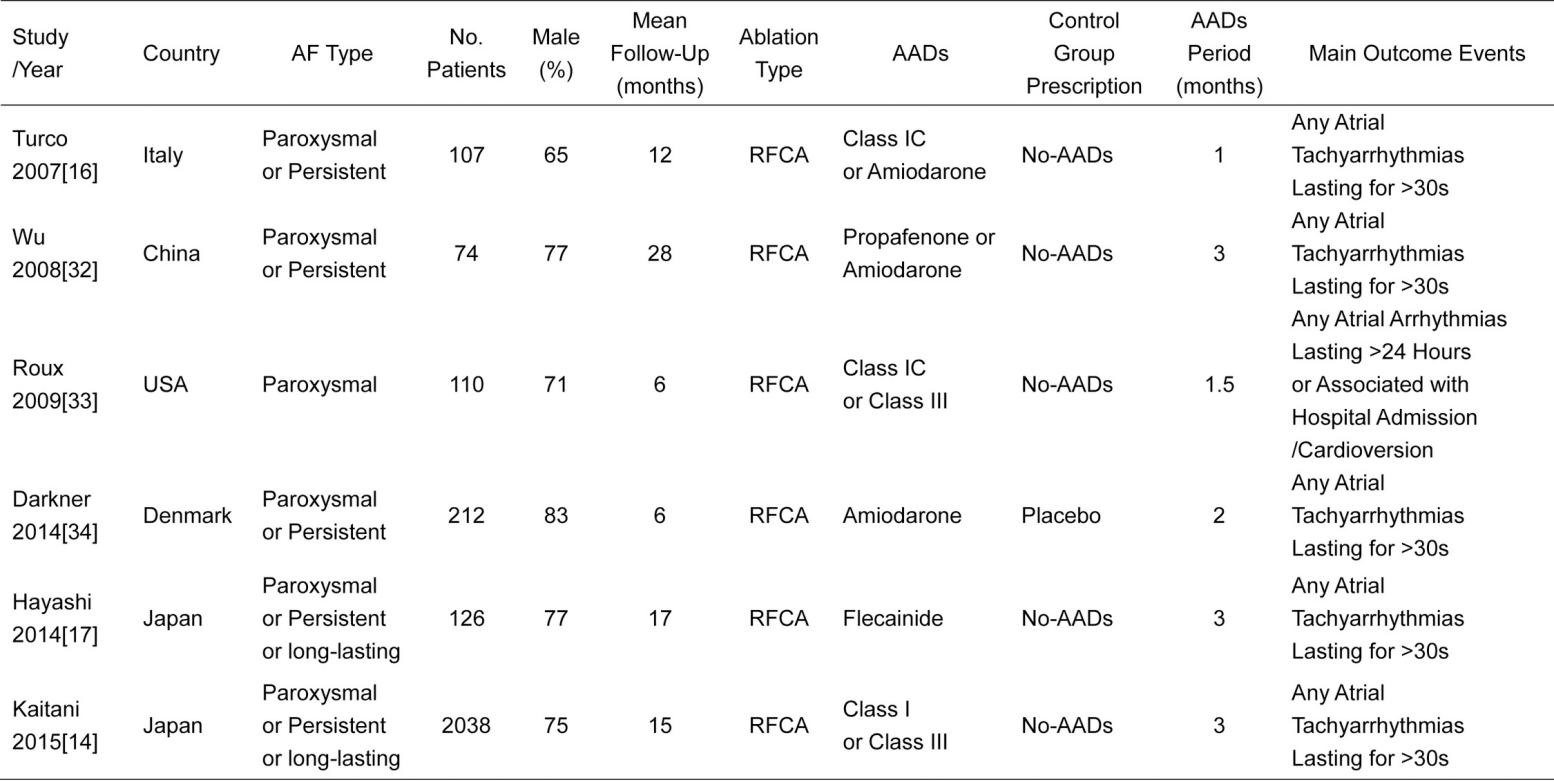
PICO characteristics of the 6 Included Randomized Controlled Trials in This Meta-Analysis. AF = atrial fibrillation. AADs = antiarrhythmic drugs. RFCA = radiofrequency catheter ablation.

**Fig 3:**
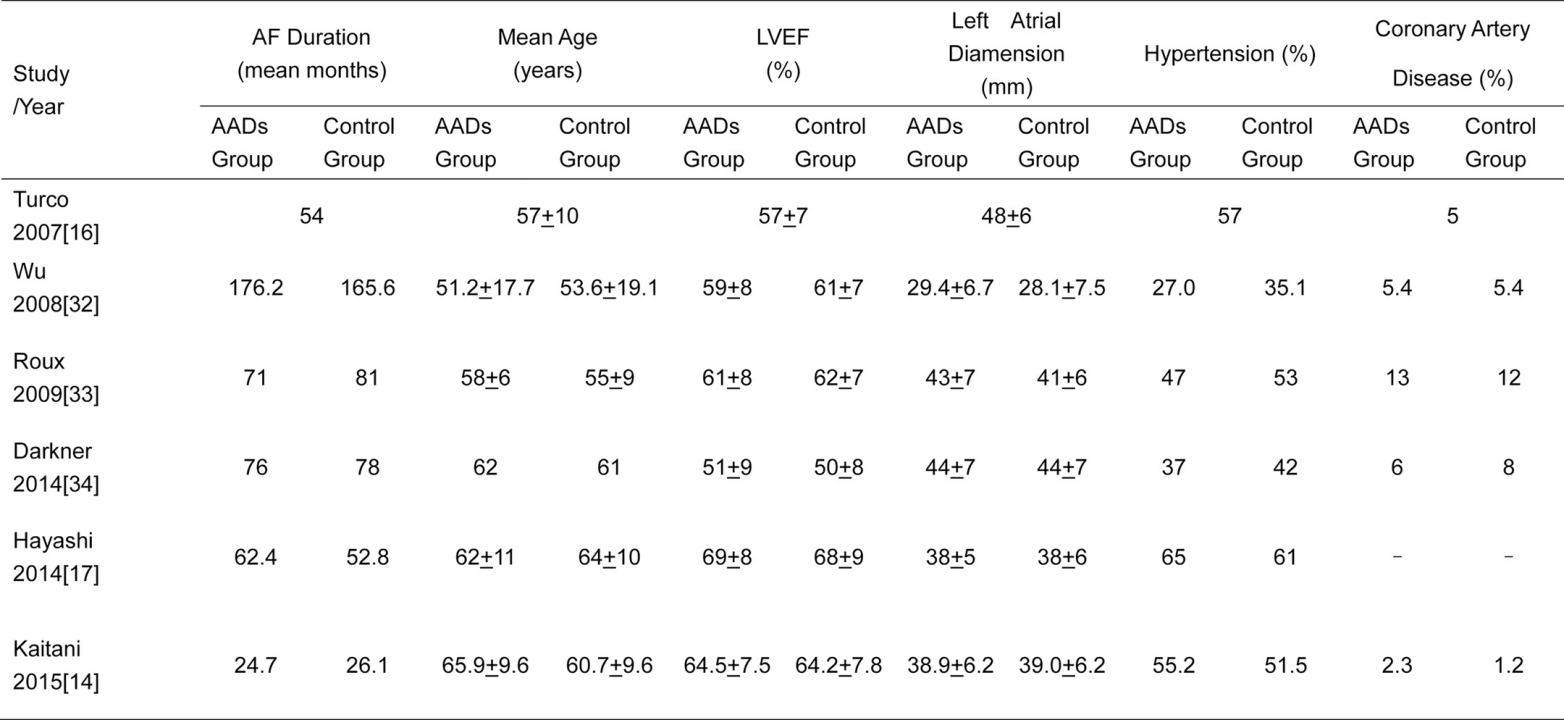
Detailed patient information of the 6 Included Randomized Controlled Trials in This Meta-Analysis. AF = atrial fibrillation. AADs = antiarrhythmic drugs. LVEF = left ventricular ejection fraction.

The main characteristics of 6 included RCTs with 2667 patients are shown in **Figs 2** and **3**. The population sizes of trials ranged from 74 to 2038. A total of 1329 patients in AADs group received short-term AADs therapy and 1338 patients in control group received no-AADs after ablation. Most of the RCTs included patients with paroxysmal and persistent AF except the trial by Roux et al[33] which only enrolled patients with paroxysmal AF. The mean AF duration of these RCTs ranged from 24 to 81 months except the trial by Wu et al[32] which included patients with more than 10 years of AF history. The mean age ranged from 51 to 65 years, and the proportion of men ranged from 65% to 83%. Most of the included patients had normal left ventricular ejection fraction (LVEF) with the baseline LVEF ranging from 50% to 69%. At baseline, mean left atrial diameter varied from 28 to 48 mm and did not differ in those who received short-term AADs therapy versus those who didn’t. Enrolled Patients had variable underlying heart diseases, while the most common comorbidity was hypertension. All enrolled patients underwent catheter ablation with PVI-based strategy. Patients in AADs group received class I or III AADs therapy for 1 to 3 months, while patients in control group received no-AADs treatment after AF ablation. And mean follow-up duration of RCTs ranged from 6 to 28 months.

### Risk of Bias Assessment

**Fig 4:**
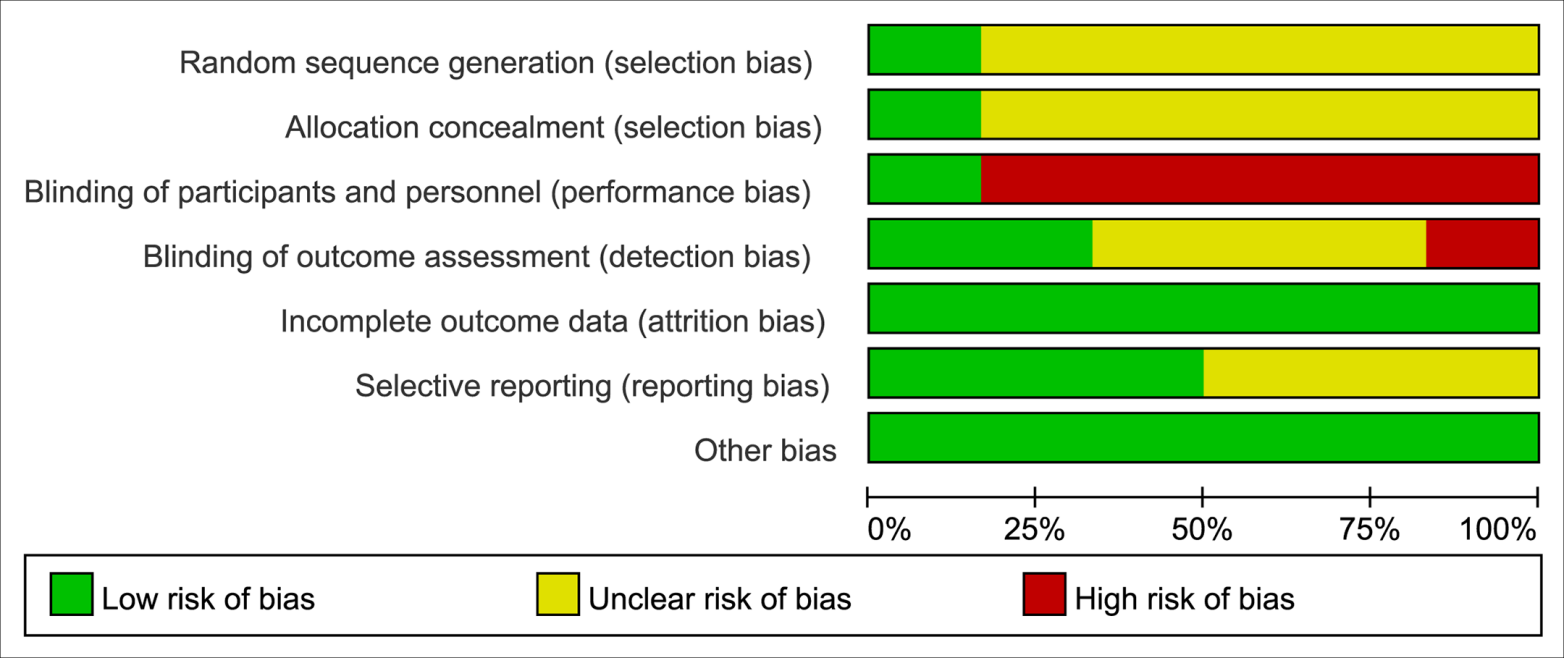
Risk of bias for this meta-analysis: judgements about each risk of bias item presented as percentages across all included randomized controlled trials.

**Fig 5:**
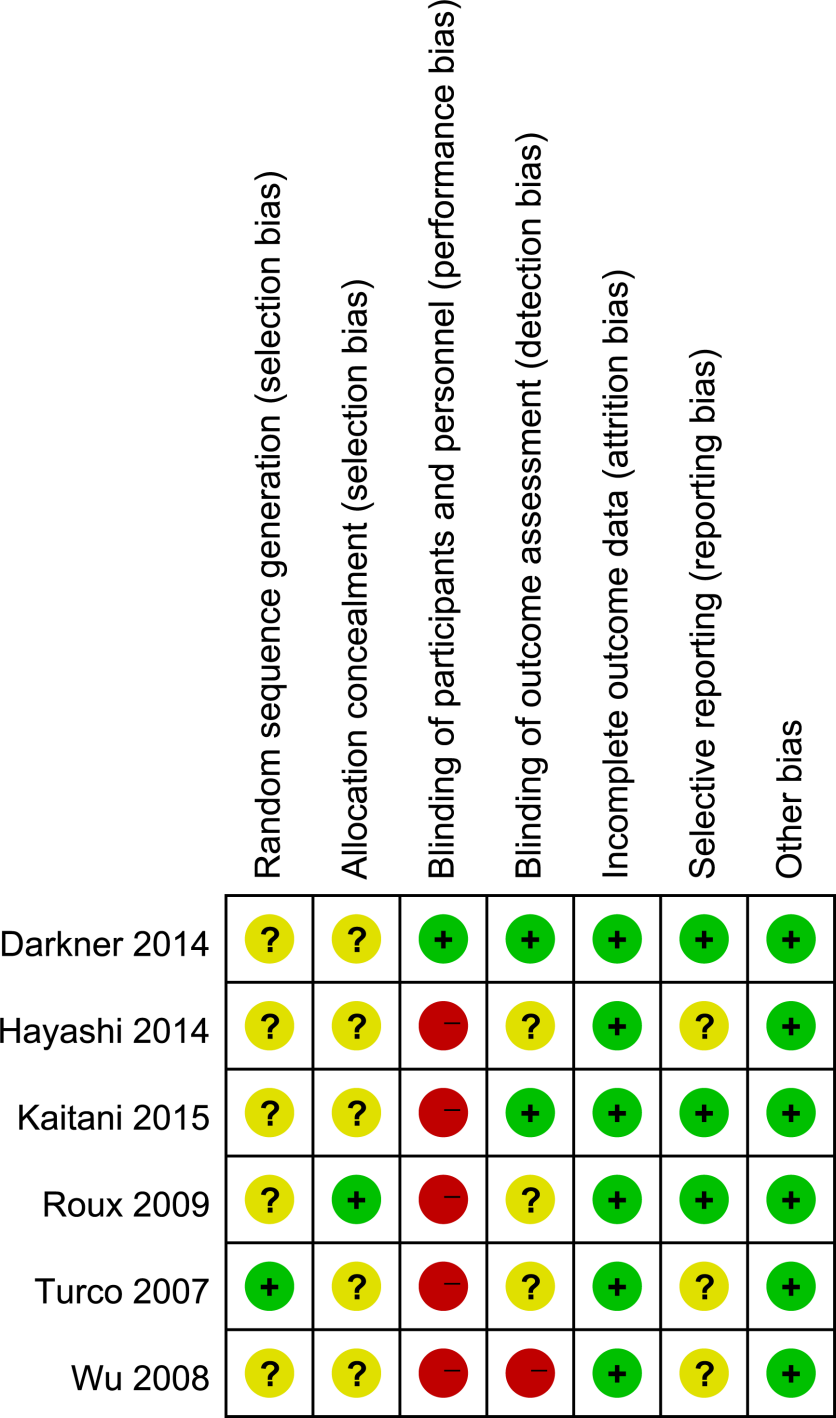
Risk of bias summary of the included randomized controlled trials: details about each risk of bias item for each included trials. Green = low risk of bias, Yellow = unclear risk of bias, Red = high risk of bias.

Details of risk of bias are summarized in **Figs 4** and **5**. According to the Cochrane risk-of-bias tool, 5 of the included RCTs are open-label studies[14,16,17,32,33] without blinding of participants and personnel, resulting in high risk of performance bias. All of the included RCTs didn’t report the information of random sequence generation except the trial by Turco et al[16]; five RCTs[14,16,17,32,34] didn’t report adequate information about allocation concealment. These problems resulted in the unclear risk of selection bias. For blinding of outcome assessment, one trial[32] was openly assessed by the outcome assessor and 3 RCTs[16,17,33] didn’t report the related information. Overall, based on the above information, one trial[34] was categorized as at unclear risk of bias, and five[14,16,17,32,33] as at high risk of bias. Therefore, the evidence level for primary and secondary outcome of this meta-analysis was partly downgraded.

### Primary Outcome

**Fig 6:**
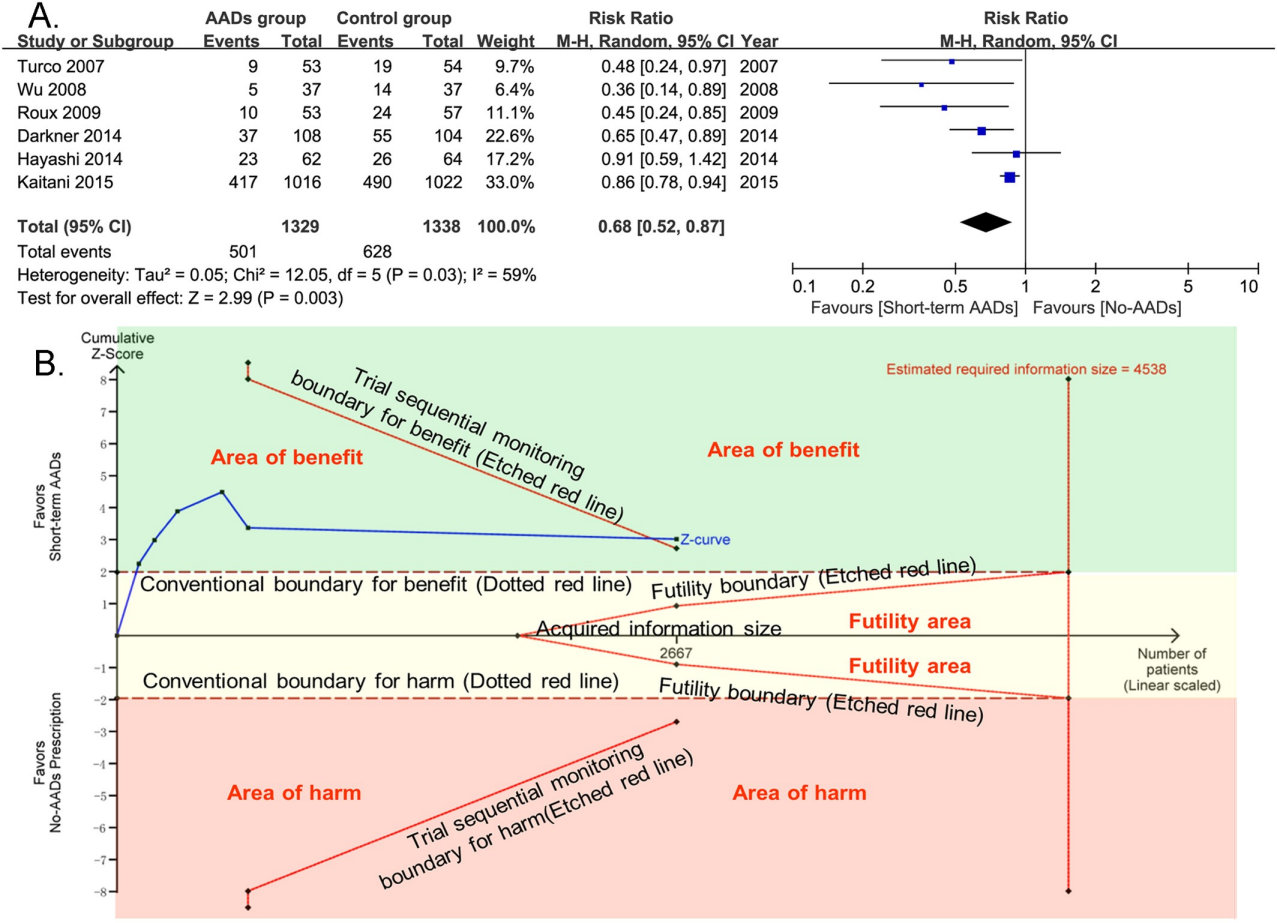
Effect of short-term AADs use versus no-AADs prescription after AF ablation on early recurrence of atrial arrhythmias. AADs = antiarrhythmic drugs; CI = confidence intervals. TSA results showed that the cumulative Z-curve crossed both the conventional boundary for benefit and the trial sequential monitoring boundary for benefit and entered the area of benefit, which established sufficient and conclusive evidence. X-axis: the number of patients included; Y-axis: the cumulative Z-Score; The red dotted lines: conventional boundaries (Z-score = 1.96, two-sided P value = 0.05); The red etched lines: trial sequential monitoring boundaries; Blue full line: the cumulative Z-curve; Vertical red etched line: the estimated required information size; The estimated required information size of 4538 patients was calculated using α = 0.05 (two sided), β = 0.20 (power 80%), an anticipated relative risk reduction of 25%, and an event proportion of 46.9% in the control group. Upper green square area: area of benefit; Middle faint yellow square area: futility area; Lower red square area: area of harm.

**Fig 7:**
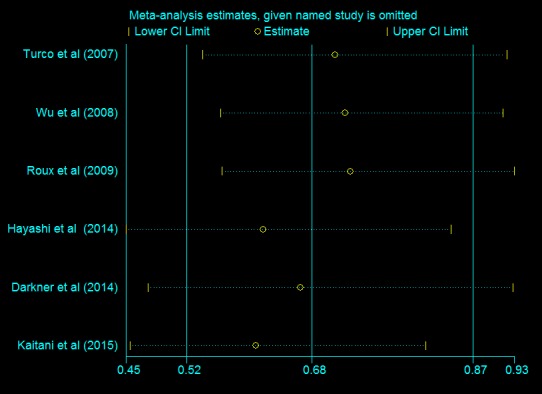
Sensitivity analysis of early recurrence of atrial arrhythmia. Single trial was excluded each time, however, pooled estimate and 95% confidence interval had no significant changes.

For the primary outcome of early recurrence of atrial arrhythmia, 6 included RCTs with 2667 patients provided the related information. Compared with no-AADs prescription in control group, short-term use of AADs after AF ablation significantly reduced the risk of early recurrence of atrial arrhythmia (RR, 0.68; 95% CI, 0.52–0.87; p = 0.003; **Fig 6A**), with moderate heterogeneity (I^2^ = 59%; p_het_ = 0.03). In consideration of the moderate heterogeneity, to evaluate the influence of single studies on the pooled estimate and the consistency of primary outcome, sensitivity analysis with consecutively excluding one single trial each time was performed. The meta-analyses after excluding every trial one at a time had no significant effect on the pooled estimate and 95% confidence interval (**Fig 7**). Moreover, potential publication bias was found by Harbord (p = 0.048) and Peters tests (p = 0.05). Despite the estimating required information size was not met (4538 patients), TSA results showed that the cumulative Z-curve crossed both the conventional boundary for benefit and the trial sequential monitoring boundary for benefit and entered the area of benefit, which established sufficient and conclusive evidence for a 25% reduction in the relative risk of early recurrence of atrial fibrillation with short-term AADs use after ablation. Based on the above TSA results, further trials aimed to evaluate the effect of short-term AADs use on prevention of early recurrence of atrial arrhythmia after AF ablation, are not required and are unlikely to alter the present conclusions (**Fig 6B**).

### Secondary Outcomes

**Fig 8:**
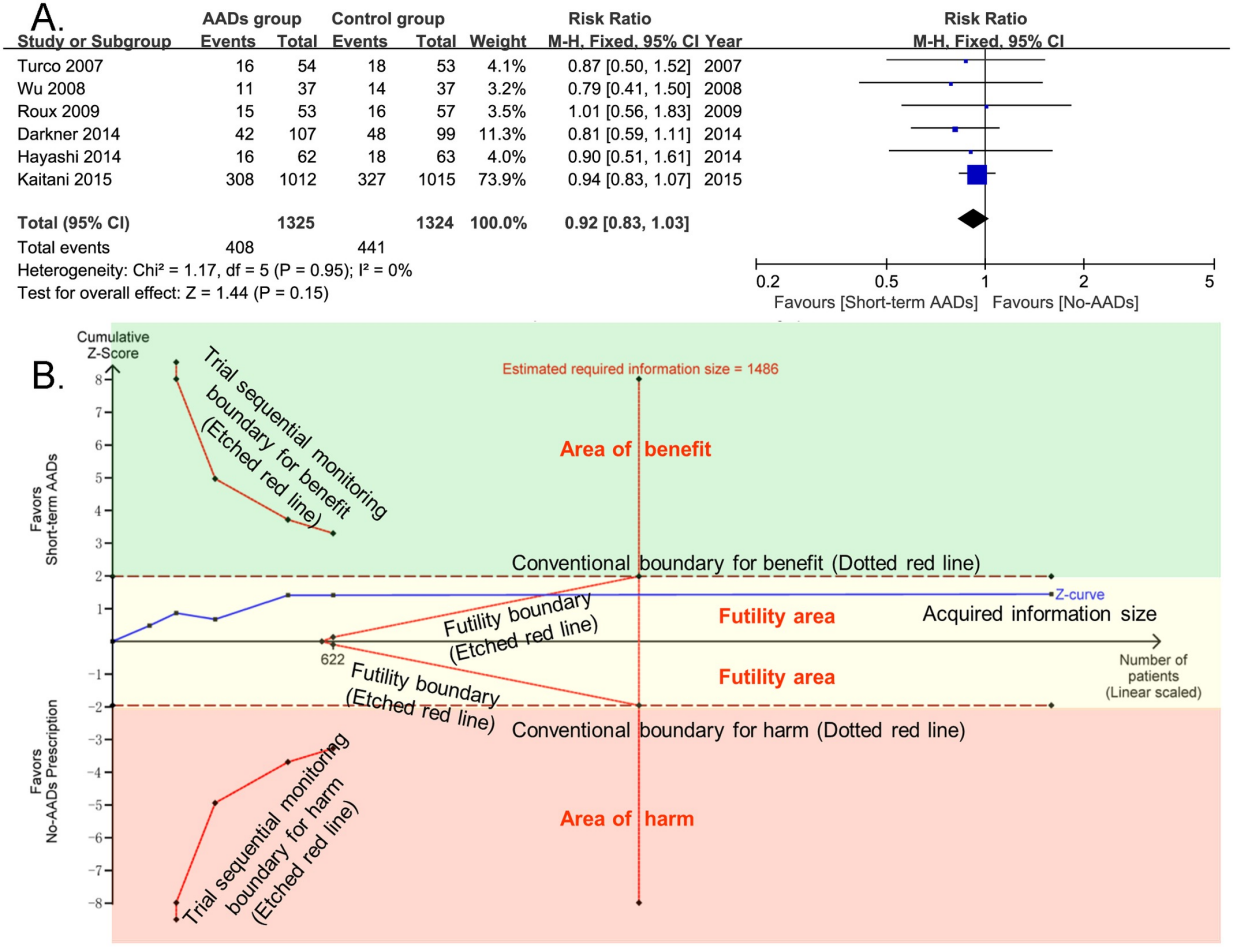
Effect of short-term AADs use versus no-AADs prescription after AF ablation on late recurrence of atrial arrhythmias. AADs = antiarrhythmic drugs; CI = confidence intervals. TSA results showed that the cumulative Z-curve crossed the futility boundary and entered the futility area, indicating the negative result was also sufficient and conclusive. X-axis: the number of patients included; Y-axis: the cumulative Z-Score; The red dotted lines: conventional boundaries (Z-score = 1.96, two-sided P value = 0.05); The red etched lines: trial sequential monitoring boundaries; Blue full line: the cumulative Z-curve; Vertical red etched line: the estimated required information size; The reached estimating required information size of 622 patients was calculated using α = 0.05 (two sided), β = 0.20 (power 80%), an anticipated relative risk reduction of 20%, and an event proportion of 33.3% in the control group. Upper green square area: area of benefit; Middle faint yellow square area: futility area; Lower red square area: area of harm.

For late recurrence of atrial arrhythmia, 6 included RCTs totaling 2649 patients provided the related information. Compared with no-AADs prescription in control group, short-term use of AADs after AF ablation didn’t significantly reduce the risk of late recurrence of atrial arrhythmia (RR, 0.92; 95% CI, 0.83–1.03; p = 0.15; **Fig 8A**), without heterogeneity (I^2^ = 0%; p_het_ = 0.95). For publication bias, both Harbord (p = 0.27) and Peters tests (p = 0.27) were not significant. TSA results showed that the estimating required information size was met with the present enrolled RCTs(1486 patients). And the cumulative Z-curve crossed the futility boundary and entered the futility area, indicating sufficient and conclusive evidence for a 20% reduction in the relative risk of late recurrence of atrial arrhythmia with short-term AADs use after ablation. The results also suggested that further clinical trials, aimed to evaluate the effect of short-term AADs use on prevention of late recurrence of atrial arrhythmia after AF ablation, are not required and are unlikely to alter the present negative conclusions (**Fig 8B**).

**Fig 9:**
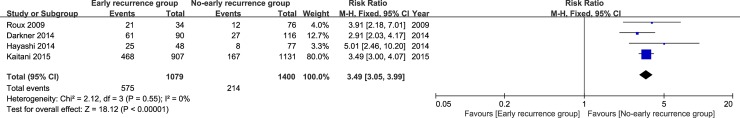
Early atrial arrhythmia recurrence after atrial fibrillation ablation is a risk factor of late recurrence of atrial arrhythmia. The pooled RR was calculated with fixed effects Mantel-Hænzel model.

Additionally, the data related to late atrial arrhythmia recurrence events between patients with and without evidence of early atrial arrhythmia recurrence were extracted from 4 included RCTs with 2479 patients. The pooled RR was calculated with fixed effects Mantel-Hænzel model. The result showed that the risk of late atrial arrhythmia recurrence was 2.49-fold higher in patients with early recurrence of atrial arrhythmia than in patients without early recurrence (RR, 3.49; 95% CI, 3.05–3.99; p < 0.001; **Fig 9**), without heterogeneity (I^2^ = 0%; p_het_ = 0.55).

### GRADE Profile Evidence

**Fig 10:**
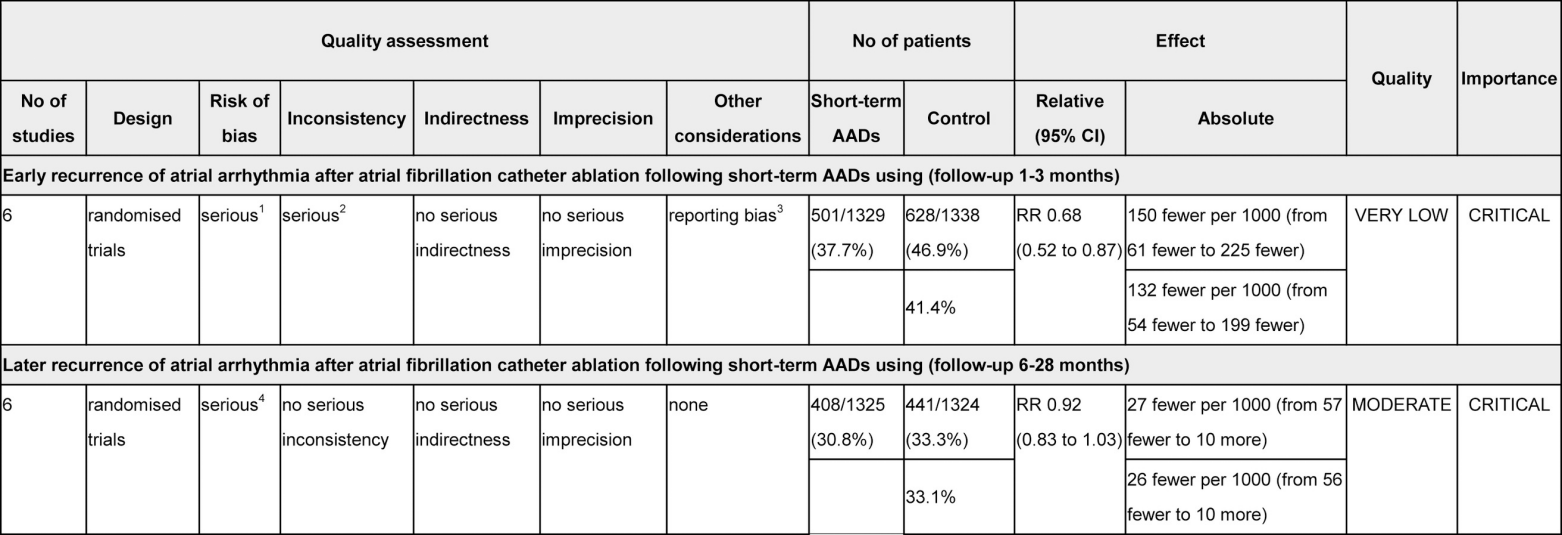
The GRADE Evidence Profile for the Primary and Secondary outcome of This Meta-Analysis. GRADE Working Group grades of evidence **High quality:** Further research is very unlikely to change our confidence in the estimate of effect. **Moderate quality:** Further research is likely to have an important impact on our confidence in the estimate of effect and may change the estimate. **Low quality:** Further research is very likely to have an important impact on our confidence in the estimate of effect and is likely to change the estimate. **Very low quality:** We are very uncertain about the estimate. ^1^ Although most of included RCTs were judged as high risk of performance bias because of without blinding of participants and personnel, the predefined objective outcome was just partly influenced. ^2^ Heterogeneity (I2 = 59%) was found. ^3^ As shown by Harbord and Peters tests, publication bias may exist. ^4^ Although most of included RCTs were judged as high risk of performance bias because of without blinding of participants and personnel, the predefined objective outcome was partly influenced.

The GRADE evidence profiles for the primary and secondary outcomes are shown in **Fig 10**. The GRADE Working Group grades level of evidence is very low for early recurrence of atrial arrhythmia (short-term AADs use versus no-AADs prescription after ablation), and moderate for late recurrence of atrial arrhythmia (short-term AADs use versus no-AADs prescription after ablation).

## Discussion

### Main Findings

The question addressed by this meta-analysis was whether short-term AADs use after catheter ablation of AF, compared with no-AADs prescription, could prevent the recurrence of atrial arrhythmia. Our meta-analysis comprehensively and systematically reviewed the current available literature, included 2667 patients from 6 RCTs, and found that (1) Short-term use of AADs compared with no-AADs prescription after AF ablation significantly reduced the risk of early recurrence of atrial arrhythmia. The evidence of this benefit was confirmed by TSA, which demonstrated this conclusion was reliable and conclusive. (2) Short-term use of AADs after AF ablation had no significant benefit on the prevention of late recurrence of atrial arrhythmia compared with no-AADs prescription in control group, which was also confirmed by TSA and the estimating required information size had been met with the present data. (3) Early recurrence of atrial arrhythmia was definitely a risk factor of late recurrence of atrial arrhythmia.

### Comparison with Published Meta-analysis

Preceding this review, one meta-analysis on this topic conducted by Xu et al[18] was published on Cardiovascular Therapeutics in August 2015. Although the main finding of our meta-analysis was consistent with the meta-analysis conducted by Xu et al[18], differences between our respective results should be noted. First, the previous published meta-analysis included 6 RCTs with a total of 814 patients. In comparison, our present meta-analysis included 6 RCTs totaling 2667 patients. Secondly, as mentioned, the included patients in the meta-analysis published by Xu et al had obvious clinical heterogeneity, as in one of the included trials by Brignole et al[19], the enrolled AF patients received atrioventricular junction ablation treatment but not PVI-based catheter ablation and in another RCT by Jun et al[20] the control group received either class I or III AADs but not placebo while the extensive AADs therapy group received both class I and III AADs. Apart from these two mentioned RCTs with clinical heterogeneity, only 4 RCTs with 555 patients in previous published meta-analysis had the appropriate clinical homogeneity for meta-analysis. In contrast, all the enrolled AF patients in our meta-analysis received PVI-based catheter ablation and the patients in control group received no-AADs prescription after ablation procedure. Third, we further applied TSA to provide more conservative estimate. The results of TSA showed that our present meta-analysis established sufficient and conclusive evidence. Last but not the least, we reported our meta-analysis in strict compliance with the PRISMA guidelines and also evaluated the quality of evidence for outcomes using GRADE to help healthcare professionals to make clinical decisions.

### The relationship between early recurrence and late recurrence

Our meta-analysis showed that although short-term AADs use after AF ablation significantly reduced the risk of early recurrence of atrial arrhythmia, it did not lead to the corresponding reduction in the risk of late recurrence of atrial arrhythmia. However, it has been shown that early recurrence of atrial arrhythmias after ablation was strongly associated with late atrial arrhythmia recurrence[14,15]. And our results also showed that early recurrence of atrial arrhythmia was definitely a risk factor of late atrial arrhythmia recurrence. Overall, the reasons to why early recurrence of atrial arrhythmia, as a risk factor for late atrial arrhythmia recurrence, can be decreased by short-term AADs use but not results in corresponding reduction of late recurrence of atrial arrhythmia, have not been intensively investigated.

The proposed mechanisms of early recurrence after AF ablation include mechanical and thermal injury provoking an inflammatory response[9,35,36], and the modification of autonomic nervous system[37,38]. Previous studies have demonstrated that the levels of inflammatory cytokines were significantly elevated after catheter ablation[35,39]. Among these cytokines, tumor necrosis factor (TNF) can directly alter Ca^2+^ handling in cardiomyocytes, which is crucial for the initiation of AF and atrial electrical remodeling[10,40,41]; platelet-derived growth factor (PDGF) can reduce the duration of action potentials and Ca^2+^ transients of cardiomyocytes[42]; and also C-reactive Protein is associated with an increased number of identified nonpulmonary vein ectopies and high-frequency sites in the left atrium[43,44]. Additionally, inflammation also increases the heterogeneity of conduction and AF duration[44,45]. Thus, elevated inflammatory cytokines can irritate the left atrium by influencing the electrophysiological characteristics of atrial myocardium and increase vulnerability to AF, which may be part of the reason for high rate of early atrial arrhythmia recurrences after AF ablation. It is known that AADs, especially the class I and III AADs, could lead to the prolongation of the effective refractory period and the action potential durations of atrial myocytes by blocking the sodium and potassium channels. Therefore, as we theoretically hypothesized, short-term use of AADs may prevent the above alteration of atrial electrophysiological characteristics through blocking ion channels and ultimately leads to reduction of early atrial arrhythmia recurrences.

The mechanisms for early recurrence and late recurrence of atrial arrhythmias are different. Previous studies indicated that late recurrences of atrial arrhythmias were mainly ascribed to incomplete pulmonary vein isolation, recovery of electrical conduction between the pulmonary veins and left atrium, or that of other block lines created in the previous procedure[1,46,47]. Moreover, the experiences of centers for catheter ablation procedure of AF were also an important factor that influenced the incidence of late atrial arrhythmia recurrences. However, all these factors can not be effectively resolved by short-term use of AADs. A recent report by Zhang et al[48] has shown that a second ablation procedure was more effective in maintaining sinus rhythm than AADs in patients with late atrial arrhythmia recurrence after catheter ablation of AF. As we above discussed, the main causes for early recurrence of atrial arrhythmias included post-ablation inflammations, temporary autonomic imbalance, and also the delay of atrial radiofrequency lesion formation[49]. Moreover, recovery of pulmonary veins-left atrium conduction was also proposed as a potential cause for early atrial arrhythmia recurrences[50,51]. However, the referred to effect of post-ablation inflammatory cytokines on early atrial arrhythmia recurrences were elevated in the days immediately following PVI but returned to baseline within 30 days[52], while significant autonomic imbalance was observed at one week following PVI and spontaneous recovered within one month[37]. It has been proposed that these transient factors would not be expected to lead to late atrial arrhythmia recurrences[50]. Previous studies have demonstrated that early atrial arrhythmia recurrences within one month following PVI, which may be promoted by these transient factors, were less predictive for late recurrences compared with that initiated in the second and third months post-ablation which may mainly be induced by pulmonary veins-left atrium electrical reconnection[53,54]. Therefore, as this meta-analysis shown, short-term use of AADs after AF ablation, through the mechanism of preventing the alteration of atrial electrophysiological characteristics induced by the above transient factors, although significantly reduced the risk of early atrial arrhythmia recurrences, but not resulted in the corresponding reduction of late atrial arrhythmia recurrences.

### Implications for Clinical Practice

Based on our meta-analysis results, in clinical practice, short-term use of AADs after AF ablation just reduces early recurrence of atrial arrhythmia within 3 months but not affect the risk of late atrial arrhythmia recurrences. For late recurrence of atrial arrhythmia, it has been suggested by RCT[48] and guidelines[1] that repeat ablation may be a preferable option. However, the reduction of early atrial arrhythmia recurrence may lead to better medical compliance of patients. Thus, pharmacologic rhythm control approach with short-term use of antiarrhythmic drugs (AADs) but not repeat ablation within 3 months after AF ablation has been proposed by the guidelines[1] and expert consensus[12].

### Limitations

Our meta-analysis also had limitations. First, this is a meta-analysis at the study level and a variety of differences exist in ablation procedures, follow-up periods, physician experience, and antiarrhythmic drugs used among enrolled RCTs. Second, most of the included trials were not blinded, which may result in performance and detection bias and influence the evidence level of outcomes.

## Conclusions

Short-term use of AADs after AF ablation can significantly decrease the risk of early recurrence of atrial arrhythmia, which is a risk factor of late atrial arrhythmia recurrence, but not lead to the corresponding reduction in the risk of late atrial arrhythmia recurrence.

## Supporting Information

S1 File PRISMA checklist. (DOC)

S2 File Lists of full-text excluded articles and the reasons for exclusion. (DOCX)

## Abbreviations

AADs: antiarrhythmic drugs
AF: atrial fibrillation
CI: confidence intervals
CRP: C-reactive protein
GRADE: Grading of Recommendations Assessment, Development, and Evaluation
LVEF: left ventricular ejection fraction
MeSH: Medical Subject Headings
PDGF: platelet-derived growth factor
PRISMA: Preferred Reporting Items for Systematic Reviews and Meta-Analyses statement
PVI: pulmonary vein isolation
RCT: randomized controlled trial
RR: relative risk
TNF: tumor necrosis factor
TSA: trial sequential analysis
