# Supplementary material for: Efficacy of Short-Term Antiarrhythmic Drugs Use after Catheter Ablation of Atrial Fibrillation—A Systematic Review with Meta-Analyses and Trial Sequential Analyses of Randomized Controlled Trials
Source: PLoS One. 2016 May 25;11(5):e0156121. doi: 10.1371/journal.pone.0156121 (PMC4880320; doi:10.1371/journal.pone.0156121)
Supplement: S2 File — (DOCX) [file pone.0156121.s002.docx]

**Lists of full-text excluded articles and the reasons for exclusion**

There are 7 studies excluded by review of full text.

**3 for not met the inclusion criteria：**

[1] Gu J, Liu X, Tan H, Zhou L, Gu J, et al. (2012) Extensive antiarrhythmic drugs after catheter ablation of persistent atrial fibrillation. Acta Cardiol 67: 407-414.

[2] Miwa Y, Minamiguchi H, Bhandari AK, Cannom DS, Ho IC (2014) Amiodarone reduces the amount of ablation during catheter ablation for persistent atrial fibrillation. Europace 16: 1007-1014.

[3] Sohns C, von GV, Sossalla S, Bergau L, Seegers J, et al. (2014) Antiarrhythmic drug therapy for maintaining sinus rhythm early after pulmonary vein ablation in patients with symptomatic atrial fibrillation. Cardiovasc Ther 32: 7-12.

**2 for not the randomized controlled trials：**

[1] Shamiss Y, Khaykin Y, Oosthuizen R, Tunney D, Sarak B, et al. (2009) Dofetilide is safe and effective in preventing atrial fibrillation recurrences in patients accepted for catheter ablation. Europace 11: 1448-1455.

[2] Igarashi M, Tada H, Sekiguchi Y, Yamasaki H, Arimoto T, et al. (2010) Effect of restoration of sinus rhythm by extensive antiarrhythmic drugs in predicting results of catheter ablation of persistent atrial fibrillation. Am J Cardiol 106: 62-68.

**2 for no available data:**

[1] Park YM, Lee DI, Park HC, Shim J, Ko KJ, et al. (2014) Effect of pilsicainide® versus other class IC anti-arrhythmic drugs after catheter ablation of paroxysmal atrial fibrillation; prospective randomized study. Heart Rhythm 11: S396-S398.

[2] Zhang XD, Gu J, Jiang WF, Zhao L, Zhou L, et al. (2014) Optimal rhythm-control strategy for recurrent atrial tachycardia after catheter ablation of persistent atrial fibrillation: a randomized clinical trial. Eur Heart J 35: 1327-1334.
